# Intestinal microbiota alterations by dietary exposure to chemicals from food cooking and processing. Application of data science for risk prediction

**DOI:** 10.1016/j.csbj.2021.01.037

**Published:** 2021-01-29

**Authors:** Sergio Ruiz-Saavedra, Herminio García-González, Silvia Arboleya, Nuria Salazar, José Emilio Labra-Gayo, Irene Díaz, Miguel Gueimonde, Sonia González, Clara G. de los Reyes-Gavilán

**Affiliations:** a Department of Microbiology and Biochemistry of Dairy Products, Instituto de Productos Lácteos de Asturias (IPLA-CSIC), 33300 Villaviciosa, Asturias, Spain; b Department of Functional Biology, University of Oviedo, 33006 Oviedo, Asturias, Spain; c Diet, Microbiota and Health Group, Instituto de Investigación Sanitaria del Principado de Asturias (ISPA), 33011 Oviedo, Spain; d Department of Computer Science, University of Oviedo, C/ Federico García Lorca S/N, 33007 Oviedo, Asturias, Spain; e IT and Communications Service, University of Oviedo, C/ Fernando Bongera S/N, 33006 Oviedo, Asturias, Spain

**Keywords:** Intestinal microbiota, Colorectal cancer, Diet, Toxic chemicals, Machine learning, Semantic web

## Abstract

Diet is one of the main sources of exposure to toxic chemicals with carcinogenic potential, some of which are generated during food processing, depending on the type of food (primarily meat, fish, bread and potatoes), cooking methods and temperature. Although demonstrated in animal models at high doses, an unequivocal link between dietary exposure to these compounds with disease has not been proven in humans. A major difficulty in assessing the actual intake of these toxic compounds is the lack of standardised and harmonised protocols for collecting and analysing dietary information. The intestinal microbiota (IM) has a great influence on health and is altered in some diseases such as colorectal cancer (CRC). Diet influences the composition and activity of the IM, and the net exposure to genotoxicity of potential dietary carcinogens in the gut depends on the interaction among these compounds, IM and diet. This review analyses critically the difficulties and challenges in the study of interactions among these three actors on the onset of CRC. Machine Learning (ML) of data obtained in subclinical and precancerous stages would help to establish risk thresholds for the intake of toxic compounds generated during food processing as related to diet and IM profiles, whereas Semantic Web could improve data accessibility and usability from different studies, as well as helping to elucidate novel interactions among those chemicals, IM and diet.

## 1 Introduction

Diet is one of the main sources of exposure to toxic compounds with carcinogenic potential. In October 2015 the International Agency for Research on Cancer from the World Health Organization (IARC-WHO) announced the classification of processed meat as “carcinogenic to humans” and red meat as “probably carcinogenic to humans” [1]. Diets from most developed countries are characterized by high intakes of meat, which is often fried, griddled or barbecued, and by an increasing consumption of processed foods. When cooking muscle meat from animals or fish at high temperature, some chemicals are formed at levels that depend on the cooking procedure and temperature; some of these compounds can cause cancer when administered at high doses in experimental animals [2]. However, although the intake of dietary compounds with carcinogenic potential in humans is considerably lower than in experimental animals, lifetime exposure can differ considerably among individuals. No regulations exist about the presence in foods of cooking -related potential carcinogens. This aspect is specially relevant for public health, as most cooking mutagen/genotoxic compounds are generated at home, restaurants and local ready-to-eat food providers.

Despite that some international projects have evaluated the association between nutrition (including cooking methods) and cancer, such as the *European Prospective Investigation into Cancer and Nutrition* (EPIC) or the NIH-AARP Diet and Health Study, an unequivocal link between dietary exposure to chemicals and human cancer [3] has not been shown. The underlying reasons for this may be as follows: i) the difficulty to determine the exact exposure to these compounds (depending not only on the intake but also on the cummulative exposure and delayed effect through life), ii) interindividual variation in the detoxifying activity of endogenous enzymes, iii) cummulative exposure to toxic compounds from different environmental sources, iv) synergistic interaction among different compounds and, v) the role, not sufficiently explored to date, of the interaction between diet and the intestinal microbiota (IM) on the net carcinogenic potential. Therefore, studies designed to explore these interactions could help to establish risk thresholds for disease as a function of dietary intake of potential carcinogens, global diet and microbiota. The present review analyses difficulties inherent to this type of studies and how Machine Learning (ML) and Semantic Web could assist in data modelling for risk assessment.

## 2 Chemicals with carcinogenic potential formed during food cooking and processing

One of the most important risk factors for the development of cancer is the exposure to dietary toxic chemicals with carcinogenic and pro-carcinogenic potential which, when consumed regularly at certain levels, can increase the risk of triggering tumorigenic processes. Nitrates, nitrites, nitrosamines (NA), heterocyclic amines (HCA), polycyclic aromatic hydrocarbons (PAH) and acrylamide, are amongst the substances with the highest carcinogenic potential. Some of these compounds are not naturally present in foods but can be incorporated (nitrates and nitrites) or generated (NA, HCA and PAH) during the processing of foodstuffs containing nitrogenous and creatine components by heat-direct exposure procedures [4]. HCA have accumulated solid scientific evidence as cancer risk factors and are the only carcinogens formed exclusively during the cooking process. Specifically, HCA show a mutagenicity index more than 1000 times higher than benzo(a)pyrene (BaP) [3]. Carcinogens may act through various mechanisms, such as chromosomal aberrations, single strand breaks and DNA adducts or oestrogenic activity [5]. Several prospective cohort studies reported mean intakes of HCA between 69.4 ng/day and 821 ng/day in European countries [6], [7] and from 49.95 ng/day to 151.9 ng/day in Chinese communities and the United States [8], [9]. The observed variability among countries and individuals may be attributed to differences in the methodology used for the assessment of potential carcinogenic chemicals and to differences in dietary patterns and cooking preferences around the world. For example, compared to the 134.5 ng/day contribution of 50 g of broiled beef (0.00269 ppm/day), one daily serving of 50 g of broiled chicken could increase the intake of HCAs (PhIP + MeIQx) by 1350 ng/day (0.027 ppm/day) [10]. Induction of tumours in the large intestine of F344 rats and C57BL/6 mice have been demonstrated during prolonged exposure (40 to 72 weeks) to high concentrations of some HCA in diet (i.e. 300 ppm/day) [2]. Although useful to demonstrate tumorigenic potential, experiments with animals are not intended to predict true human cancer incidence associated with exposure to chemicals.

PAHs are found in cured and processed meat and fats, primarily [11]. Dietary exposure levels ranged from the order of ng/day in some Asian publications [12] to the order of μg/day reported in other publications [13]. BaP is the most-used marker to detect the presence of PAHs in foods [14], [15]. NA are detected in cured meat and smoked foods and are also endogenously formed from the interaction of nitrosating agents with amines and amides [16]. The intake of NA showed unclear relationships with pancreatic-cancer but positive associations with colorectal cancer (CRC) and gastric cancer [17], [18].

Nitrates and nitrites are often used as food additives in processed meats, fish, cheese, and fermented products, to preserve them from microbial alteration [19]. The simultaneous presence in certain foods of amino acids can lead to a chemical reaction that results in the formation of NA, especially when a heat treatment is applied; N-nitrosopyrrolidine (NPYR) and N-nitrosodimethylamine (NMDA) are the NA most frequently found in foods [19]. Several studies have shown an increased risk of CRC development for NMDA intakes of 0.03–0.07 µg/day [20].

Acrylamide is formed by asparagine decarboxylation in the presence of reducing sugars during nonenzymatic browning (Maillard reaction) [21]. It is naturally found in foods, but can also form during the thermal treatment. In European countries, the major sources of acrylamide are potatoes, coffee and cereal products [22]. Acrylamide has been classified by the EFSA [23] as probably carcinogenic to humans. However, there is still no regulation on the maximum recommended intake albeit there is a general recommendation to limit its consumption.

## 3 Challenges to determine the actual intake of toxic chemicals with carcinogenic potential generated during food cooking and processing

Recent meta-analyses of epidemiological studies are still not completely conclusive about the relationship of the intake of toxic compounds with carcinogenic potential resulting from food processing and cancer development [3] as it is complex to disentangle the effect of these compounds from the effect of the food itself. Most of the research revealing the impact of red and processed meat consumption in the relative risk of developing several chronic pathologies, such as CRC, prostate or lung cancer is the result of longitudinal epidemiological studies. Although these studies are useful from a descriptive point of view and for the generation of research hypotheses, they have a limited potential for the establishment of cause-effect relationships, leading to the continuing debate about the health impact of meat intake.

A major difficulty in assessing quantitatively the actual intake of food potential carcinogens in the population is the selection of the most appropriate method for the collection of dietary data. The food frequency questionnaire (FFQ), multiple day food records and 24-hour dietary recall are among the most extensively used tools for this purpose. With independence to the systematic and random errors inherent to these methods [24] some factors such as the time period covered by the dietary questionnaires and the number of items included or the quantification of the portions consumed, affect the quality of the information collected and therefore the conclusions drawn. It is important to note that the risk of developing cancer from exposure to environmental factors, including diet and lifestyle, is cumulative over a subject's lifetime. For this reason, it seems more appropriate to use questionnaires with the capacity to describe long-term dietary habits, such as the FFQ. However, the FFQ has the disadvantage of providing less accurate information on energy and nutrient intake compared with the other methods mentioned above. In addition, some of the postulated mechanisms linking meat consumption to cancer risk include the content of these foods in HCA [4], PAH and other compounds generated during the high-temperature processing of foods, particularly in meats cooked at “well-done” degree [4]. Therefore, at the time of quantifying the intake of different toxic compounds with carcinogenic potential, it is important to detail in a harmonised way some characteristics related to the culinary preparation of foods, such as cooking time, processing method, temperature or degree of browning [11]. This is a strong add-on difficulty because it prolongs the duration of the baseline questionnaires, increasing the number of items included. In addition, the analysis of the information obtained is more complex than usual for the calculation of a nutrient, since for each of the foods surveyed, the type of processing (preservation or cooking) and the duration and temperature of cooking should be considered. The estimation of dietary compounds with carcinogenic potential can be extracted from information compiled in various databases. The most widely used databases are those developed by the EPIC study for the European population [25] and by the Computerized Heterocyclic Amines Resource for Research in Epidemiology of Disease (CHARRED) database for the United States [26]. Both databases provide key information for integrating the analysis of dietary potential carcinogens on a systematic basis. The EPIC database compiles information obtained from 139 references regarding the content per 100 g of food in NA, HAC, PAH, nitrites and nitrates in more than 200 food items. The food composition table is classified according to the preservation method, cooking method, degree of browning and temperature [25]. This information is also present in the CHARRED database, which has developed a special module within a FFQ in conjunction with the mutagens database to estimate intake of the mutagenic compounds in cooked meats [26]. In adittion, acrylamide content was estimated from the EFSA categorisation of European food products for monitoring purposes [27].

A broader approach is necessary in the future in order to lay the foundations for improving the understanding of the complex diet-cancer association in the long term. This approach would require consensus on standardised and harmonised protocols for collecting dietary information, classifying the degree of cooking and calculating carcinogens derived from food processing. This method should be complemented with advanced tools for mathematical analysis of data that enable researchers to both identify risk factors for these pathologies and explain their impact in the complex context of a subject's global diet and lifestyles.

## 4 Intestinal microbiota and human health. Methods to study composition and functionality

The IM is defined as the set of microorganisms inhabiting the intestine. The microbiota has co-evolved with the host over thousands of years, leading to the establishment of a mutually beneficial microbiota-host relationship. The number of microorganisms in the human gut exceeds 10^14^ and this microbiota encodes a collection of genes ~10 times greater than these encoded by the human genome, providing exclusive capabilities and functions essential for the maintenance of health. The role of the IM begins in early life, participating in the development of the host́s immune, digestive and nervous systems by strengthening intestinal epithelium integrity and gut barrier, protecting against pathogens and playing a major role in helping to harvest nutrients and energy from our diet. Therefore, the IM results in a key player for host physiology [28].

This IM represents a large factory producing bioactive compounds and participating in the host́s metabolism and nutrition. Actually, host metabolism is the combination of the capabilities of both the human and the IM genomes. The microbiota ferments indigestible complex carbohydrates and proteins from the diet producing short-chain fatty acids, primarily acetate, propionate and butyrate, which are quickly absorbed by the gut epithelial cells [29]. Acetate is primarily delivered to peripheral tissues for use as a substrate in the synthesis of cholesterol and fatty acids; propionate is absorbed in the liver and participates in gluconeogenesis; and butyrate is used as one of the main energy sources by colonocytes. Other metabolites are also produced by the IM such as branched chain fatty acids, secondary bile acids, amino acids, trimethylamine, neurotransmitters, and some essential vitamins [30], [31]. Some of these metabolites may suffer further transformations, such as the case of trimethylamine which, upon absorption will be oxidised in the liver to trimethylamine-N-Oxide, a known risk factor for cardiovascular disease. Therefore, all these metabolites participate in the host’s physiology and strong evidence now supports the role of the IM in the maintenance of human homeostasis. For this reason, adverse changes in the gut microbiota composition and/or function, the so-called *dysbiosis*, are related to different gastrointestinal disorders, such as diarrhoea, inflammatory bowel disease, cancer, or extra-intestinal diseases such as obesity, allergies, neurological sicknesses or other metabolic diseases. Different stressors, including dietary changes, antibiotic or other drugs treatments, and carcinogens from the diet can be involved in the development of dysbiosis.

Members of Bacteroidetes and Firmicutes phyla followed by Actinobacteria, Proteobacteria and Verrucomicrobia primarily make up the composition of the adult IM. However, at lower taxonomical levels, the complexity of the IM is higher and is represented by thousands of different microbial species. This diversity also occurs among individuals, making almost impossible the definition of a *normal* or *healthy* IM composition for an entire population. However, it is also known that the IM exhibits high functional redundancy, meaning that some functions may be conferred by multiple bacteria, from related and unrelated species, making the IM more conserved at the functional than at compositional level [32]. Accounting for this variability, some authors have tried to define the “normal or healthy” IM as the “intestinal microbial community that assist the host to maintain a healthy status under certain environmental conditions” [33] understanding that under different environmental conditions including dietary habits the optimal microbiota for health may also be different. For this reason, when we aim to assess the effect of a specific factor or a specific disease on the gut microbiota, it is crucial to identify the specific alterations present in the gut microbiota composition but also on its functional properties, as well as the underlying mechanisms.

Human faeces constitute in practice the biological samples from which the DNA, RNA and proteins are extracted in most cases to study the intestinal microbiota composition and function whereas metabolites and other chemical compounds can be extracted as well to analyze molecules produced by the microorganisms. Currently, the study of the IM involves using the new *omics* techniques based on high-throughput sequencing tools, also called *second-generation sequencing technology*. The DNA sequencing of the whole IM and the gene functions classifications are performed by *metagenomics*. *Proteomics* sequence the protein structures to determine cell metabolism through the activity of the cell enzymes. The analysis of molecules produced by bacterial metabolism is made by *metabolomics,* and *transcriptomics* studies the complete RNA molecules quantifying the dynamic expression of genes under different conditions. The effects of gut microbiota on the host are reflected in different aspects and the combinations of those *multi-omics* tools provide a new phase in the study of the IM and its physiological role, linking the composition of the IM with host metabolism, disease pathogenesis and predictions of therapeutic targets [34].

## 5 Intestinal microbiota dysbiosis is associated with colorectal cancer and pre-cancerous states

Several studies have demonstrated that IM profiles from CRC patients are different from that of healthy individuals [35]. Generally, patients with CRC have decreased microbial diversity in faeces [36] and at the intestinal mucosa level [37]. It is currently not possible to define a common cancer-associated microbiota [11], [38]. However, although no individual member of the gut microbiota alone is sufficient to promote CRC, certain microbes have been associated with this type of cancer through the formation of harmful metabolites and the regulation of certain miRNAs, which then promote an oncogenic microenvironment. There is evidence of IM associations with CRC for *Streptococcus bovis*, which has been renamed *Streptococcus gallolyticus*, *Fusobacterium nucleatum*, *Bacteroides fragilis*, *Enterococus faecalis* and certain pathogenic strains from *Escherichia coli* [36]. However, it is not clear at present if these microorganisms are drivers or passengers in CRC. In addition, although some microbiota profiles have been associated with the onset and early progression of CRC, studies in this field are still scarce [39], [40]. Some members of the gut microbiota can produce microbial genotoxins such as colibactin by *E. coli* group B and fragylisin by *B. fragilis*. Other compounds with cytotoxic action, and potential involvement in the development of CRC are produced by intestinal microbes such as *Salmonella enterica*, *Helicobacter pylori*, *F. nucleatum*, *B. fragilis*, *Pseudomonas aeuroginosa*, *Peptostreptococcus anaerobius* and *E. faecalis* among others [11]. The microbial dysbiosis can also induce changes in host gene expression, subsequently favouring the development of CRC.

## 6 Role of the intestinal microbiota on the genotoxic/mutagenic potential of dietary toxic compounds

The genotoxicity is the capability to cause damage to the cellular genetic material, and more specifically mutagenicity is the capacity of genotoxic compounds to alter the DNA sequence, modifying the expression and functionality of genes. The genotoxicity and/or the mutagenicity in faeces could be determined in an affordable way using some *in vitro* tests currently available [11].

It has been suggested that there is an association of inflammation with the faecal genotoxicity and CRC through the relationship existing between the gut microbiota and the innate immune system [38]. Early intestinal mucosal damage (dysplastic lesions, aberrant crypt foci, and/or intestinal polyps) can precede in years the development of CRC and these mucosal lesions could be considered early markers of risk for the development of CRC. Intestinal mucosal lessions are routinely examined for diagnostic purposes in patients submitted to colonoscopy at hospitals, allowing to differentiate neoplastic lesions, preneoplastic lesions and healthy intestinal mucosa.

The efficiency of endogenous mechanisms of detoxification in the human body largely depends on the metabolic state of the host, and the type and levels of toxic compounds. Orally ingested toxic compounds initially reach the liver by direct gut wall absorption where they are detoxified through phase I (cytochrome P450 system) and phase II (sulphate, glutathione or glucuronide conjugates) enzymes and are subsequently stored in the gallbladder. Liver-generated detoxified potential carcinogens are poured again through the intestine by enterohepatic circulation during digestion (phase III) where they can be transformed by the gut microbiota.

Faecal toxic compounds contributing to genotoxicity may have diverse origins. As commented before, some members of the IM can produce endogenous metabolites with genotoxic potential. Other compounds are formed endogenously by the metabolic activity of intestinal bacteria on dietary constituents such as nitrates, dietary amines and cholesterol, or are synthesized from precursors of the human metabolism such as the N-nitroso compounds, fecapentaenes, long-chain fatty acids and secondary bile acids. The production of these toxic compounds by the IM will depend not only on the microbiota itself but also on the host physiology, and the interaction of the IM with diet. In addition, other toxic substances arriving to the gut are of exogenous origin (foods) and include mycotoxins, plant glycosides, food additives, and the chemical compounds formed during cooking and food processing commented on previously.

Studies using *in vitro* and *in vivo* models indicate that toxic dietary compounds, apart from their direct effect, could adversely affect the gut microbiota, modifying its diversity, composition and/or functionality, and affecting host-immunity and metabolism [35], [41], [42]. The IM can also modify the toxicity of these compounds by i) decreasing their toxicity through direct binding with the microorganisms and elimination with faeces, ii) metabolising and transforming them into less toxic compounds, iii) metabolising and transforming them into more toxically active molecules, and iv) interfering with detoxifying mechanisms of the host, thus exacerbating their toxicity [11]. The most notable of these last interactions is that occurring during enterohepatic circulation when toxic molecules inactivated in phase II by conjugation to glucuronides in the liver, return to the intestine by enterohepatic circulation. There, the intestinal microbial glucuronidases, mostly from Enterobacteria, *Clostridium* and *Bacteroides* members, release the inactivated chemical compound from the glucuronide and subsequently turn it back into a toxic molecule.

Global diet modulates the composition and functionality of the IM, influencing the way in which this microbial community interacts with dietary toxic compounds and with detoxifying mechanisms of the host, then contributing to increase or decrease in the intestinal toxicity. In this scenario, it would be possible to identify early shifts in microbiota patterns (composition and/or functionality) associated at variable degree with increased intestinal toxicity, the intake of chemicals with carcinogenic potential and global diet. These modifications of the microbiota (even when they could represent adaptive processes) may be associated with abnormal changes of the intestinal mucosa that would represent an augmented risk for the subsequent development of CRC. The diversity of chemical structures of dietary toxic compounds and the difficulty to determine accurately their intake with diet substantially increase the challenge of teasing out individual chemical class influences on CRC. However, initial effort like those focusing on a specific and defined group of compounds, as those chemicals generated during food processing, would make the task more realistic and affordable. These compounds could be assessed by means of dietary interviews that include cooking/preparation procedures, duration and temperature of the process, and the use of specific food composition databases.

Our hypothesis is that beyond differences in genetic susceptibilities, metabolic states and the inherent variability of microbiota profiles among individuals and human groups, the net exposure to dietary molecules with carcinogenic potential will depend on the type of compound, doses, frequency of consumption and lifetime exposure. These factors will be modified by food preparation procedures, which will be closely related to the amount of compound ingested, the global dietary patterns and IM profile of subjects. Therefore, risk thresholds for CRC could be established as a function of gut genotoxicity, IM and diet (global dietary patterns and toxic molecules intake), considering precancerous or cancerous mucosal changes as an outcome variable.

**Fig. 1:**
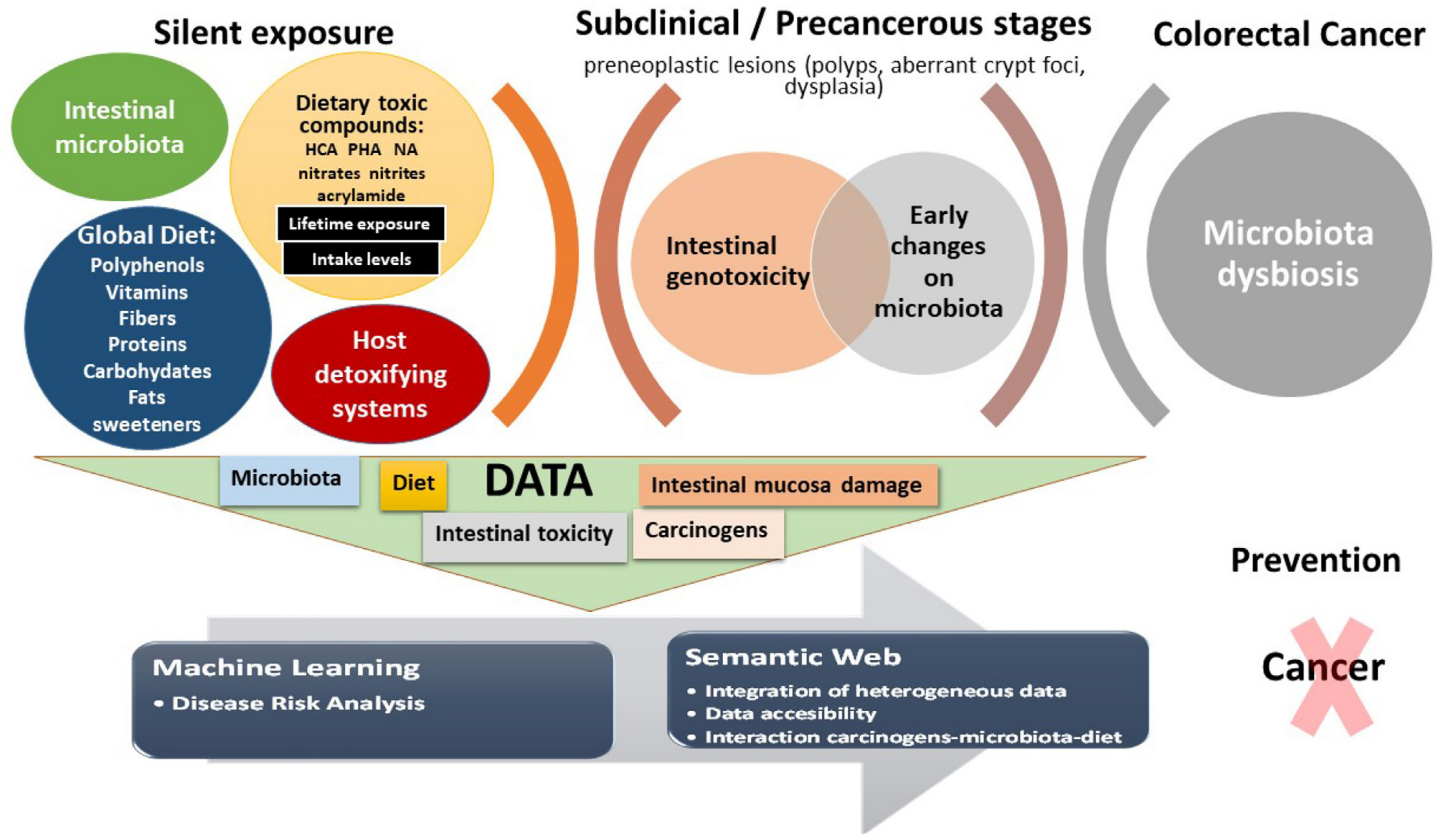
Schematic representation of risk assessment by exposure to dietary toxic compounds formed during food cooking and processing as a function of the IM, diet and intestinal toxicity, applying ML and Semantic Web. The net exposure to toxic compounds depends on the intake and time of exposure and this influences the genotoxicity at the intestinal environment. IM and global diet could modify the resulting toxicity of dietary chemicals. Prolonged exposure to high intestinal toxicity levels could lead to changes in the intestinal mucosa that may be accompanied by shifts in the intestinal microbiota. Applying ML to dietary and microbiota data in silent, subclinical and precancerous stages of intestinal mucosal damage could assist in CRC risk assessment whereas Semantic Web will facilitate data accessibility and management.

ML and Semantic Web are important tools that could assist in the treatment and modelling of such data in order to categorize the risk (Fig. 1).

The identification of changes in the microbiota associated with the intake of toxic compounds with carcinogenic potential could be useful to elaborate guidelines for food processing and dietary recommendations.

## 7 ML: a tool to assess risk by dietary exposure

ML can be considered a branch of artificial intelligence, as it attempts to use computers to complement human intelligence [43]. ML has become an essential tool for biomedical research and the modern healthcare system, given that the amount of medical and biological data requiring analysis has increased abruptly in the last years, and some ML methods have shown their ability for solving complex problems.

A key objective of any learning algorithm is to build models with good generalization capability [44]. Thus, the classification procedure is a cornerstone in any predictive problem. In addition, there is not a standard classification method to date. Different methods could be applied to design the prediction model. A decision tree (DT) is a mathematical tree where the internal nodes are tests on the variables that define the inputs and the leaf nodes are classes. C5.0, C4.5, CART or Random Forests (RF) are examples of this kind of ML. Lazy learners such as k-Nearest Neighbours (KNN) are based on learning by comparing a given test example with each training example. Artificial Neural Networks (ANN) are inspired in biological neural networks. Kernel methods as Support Vector Machines (SVM) are based on the idea of embedding the data into a high dimensional feature space using the kernel [45].

ML has been applied to dietary studies and for deciphering the effect of the exposure to pollutants and carcinogens. Thus, Chatterjee et al. [46] identified potential risk factors for preventing obesity using a broad set of different ML techniques. In another work [47] the mutual interactions between diet, microbiota, metabolic responses and the immune system were developed using a ML-based method. In a similar way, we employed DT to study the interactions between serum free fatty acids and faecal microbiota [48]. Gut microbiota was also identified as a factor in predicting personalised postprandial glycaemic response to real-life meals, obtaining an accurate prediction with boosting DT [49]. An oral malodour classifier was developed as a function of the oral microbiota in saliva, with SVM, ANN and DT, and SVM being the most accurate [50]. The decline of *Akkermansia muciniphila* was identified as a common dysbiotic marker linked to disease status by using DTs [51]. Cammarota et al. [52] recently highlighted the importance of the gut microbiome and the need of applying ML to analyse the considerably quantity of complex health care data in cancer research.

**Fig. 2:**
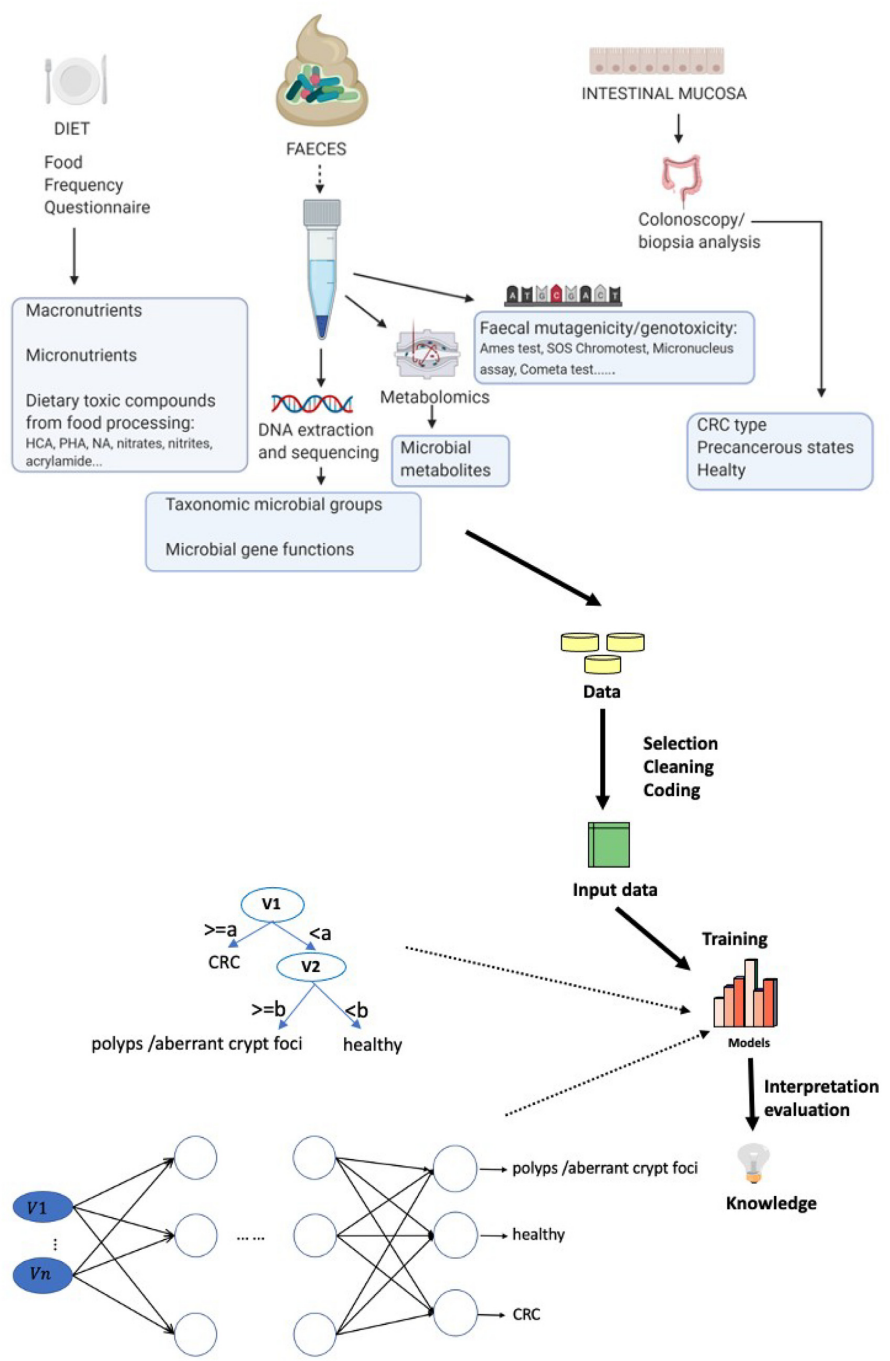
General workflow of a Machine Learning process for CRC risk assessment as a function of diet, microbiota and intestinal genotoxicity. Data from diet (FFQ), microbial metabolites, microbiota composition, microbial gene functions, and genotoxicity/mutagenicity (faeces) and biopsia analyses of the intestinal mucosa (routine colonoscopies at hospitals) are collected in a joint database and submitted to a ML process. Some ML models (such as DT, on bottom-left) allow establishing profiles and thresholds related to the input variables, while others (such as ANN, on bottom-right) are more difficult to interpret but are successful predictors.

Therefore, ML has proven to be an efficient tool to identify some key factor relationships associated with diet, health parameters and lifestyles with the microbiota and disease [48], [49], [50], [51]. Although no general rule exists *a priori* indicating which ML method is the best, depending on a given problem, it is expected that ML could successfully contribute to establishing risk thresholds for CRC as a function of the intake of chemicals with carcinogenic potential, global diet, intestinal genotoxicity and shifts in microbiota profiles. In summary, ML is able to consider factors from different sources (such as those related to ingested of potential carcinogens, diet and IM), select the most relevant ones and use them to predict the risk of CRC. A general workflow of the process is provided in Fig. 2.

## 8 Worked example of a ML process for CRC risk assessment

Since real data on diet, intake of toxic chemicals, intestinal microbiota and fecal genotoxicity/mutagenicity are not yet available in a single database, a conceptual design is proposed using previously published variables corresponding to the metabolism of healthy people and people with CRC.

*Dataset.* The dataset employed is a subset of the Colorectal Cancer Detection Using Targeted Serum Metabolic Profiling experiment from University of Washington. These data are available at https://www.metabolomicsworkbench.org/.

The dataset is composed by 234 individuals and 124 variables. For this example, Diagnosis is the target variable, that is recoded as a binary variable representing if each example presents colorectal cancer or not. From the total of existing variables in the repository, we have selected those that could be directly correlated with the diet (sugars, aminoacids, fatty acids and other compounds of interest) and including some anthropometrical variables related with diet and health, as the BMI. In addition, from the 124 variables, we have selected the following as predictive ones to run this example: “Acetylcholine” “Alanine” “Asparagine” “Aspartic_Acid” “Biotin” “Glutamic_acid” “Glutamine” “Histidine” “Linolenic_Acid” “Lysine” “Methionine” “MethylSuccinate” “Pyruvate” “Tryptophan” “BMI”

The following tables show basic statistics for these variable set depending on the value of the target variable.

**Table:** 

	Healthy		
Variable	min	mean	max
Acetylcholine	227140.38	1944056.93	3933866.8
Alanine	4029094.49	6339425.03	10736506.9
Asparagine	446544.10	697142.28	926673.2
Aspartic_Acid	367199.83	1207280.26	2736972.2
Biotin	70262.10	134817.68	218108.1
Glutamic_acid	696905.02	2101133.59	4333471.5
Glutamine	23520246.16	32222162.15	42570355.8
Histidine	10280560.84	18694498.58	29272649.1
Linolenic_Acid	403397.25	865422.94	1610396.2
Lysine	5435894.46	10117619.23	13735189.6
Methionine	306713.00	732652.67	1004676.9
MethylSuccinate	801214.57	1303371.84	1856837.4
Pyruvate	55107.82	174507.45	429810.3
Tryptophan	501607.00	3715594.49	5471963.8
BMI	20.00	27.58	42.0

**Table:** 

	Colorectal Cancer		
Variable	min	mean	max
Acetylcholine	712642.00	1755303.59	3723973.0
Alanine	2910976.71	5640811.77	9555174.6
Asparagine	456356.62	656879.58	1052985.7
Aspartic_Acid	377375.66	1636515.34	4411499.3
Biotin	63989.62	123128.62	228928.5
Glutamic_acid	916836.31	2683576.11	6559485.0
Glutamine	16182419.38	29168842.58	36269190.5
Histidine	8189632.57	14905491.46	25936858.0
Linolenic_Acid	167055.75	662328.07	1213540.7
Lysine	5237148.72	8703904.55	12749510.4
Methionine	338104.80	617976.05	1045772.3
MethylSuccinate	825623.96	1207703.72	1885528.9
Pyruvate	64219.18	199196.83	458775.4
Tryptophan	1785060.16	3451357.71	5410601.2
BMI	17.00	25.35	32.0

*Preprocessing*. As it is well known that some ML methods are quite sensitive to variable scale, continuous variables were normalized. In addition, missing values were treated using K-nearest neighbor imputation.

*Classification and evaluation*. As it was highlighted before, a key objective of any learning algorithm is to build models with good generalization capability, which is equivalent to look for models that accurately predict the class labels of previously unknown examples. Therefore, the classification procedure is a cornerstone in any predictive problem. In addition, there is no a standard classification method so far. Thus, several different methods were tested to select the one performing the best for this task, taking into account the trade-off between performance and interpretability. The methods considered in this worked example are a tree based method (C4.5), a lazy learners (Knn), a Neural Network (in particular, multilayer perceptrons, MLP) and a support vector machine with radial kernel.

Training a ML method is as complex as necessary to avoid overfitting and to correctly optimize the different hyperparameters associated to each method. In this case we have applied cross-validation with 10 folds. During the cross validation process, the specific parameters associated to each method have been optimized using the default configuration.

**Table:** 

	Sensitivity	Specificity
J48	0.75	0.63
SvmRadial	0.80	0.62
*Knn*	*0.87*	*0.87*
MLP	0.71	0.64

From the results obtained, it is clear that the method performing better according to both Sensitivity and Specificity is KNN. The value of k was 9. Note that this parameter is set experimentally in training phase. It is well known that KNN does not provide information about the features providing this classification. Thus, using this method, it is only possible to predict if an example is labelled as Healthy or having CRC. The same occurs with MLP and SvmRadial. As a consequence, if one is interested in analyzing the factors helping in the prediction, a model based on decision trees should be selected. The one employed here is C4.5. In this example, the model produced is the following:

**Figure:**
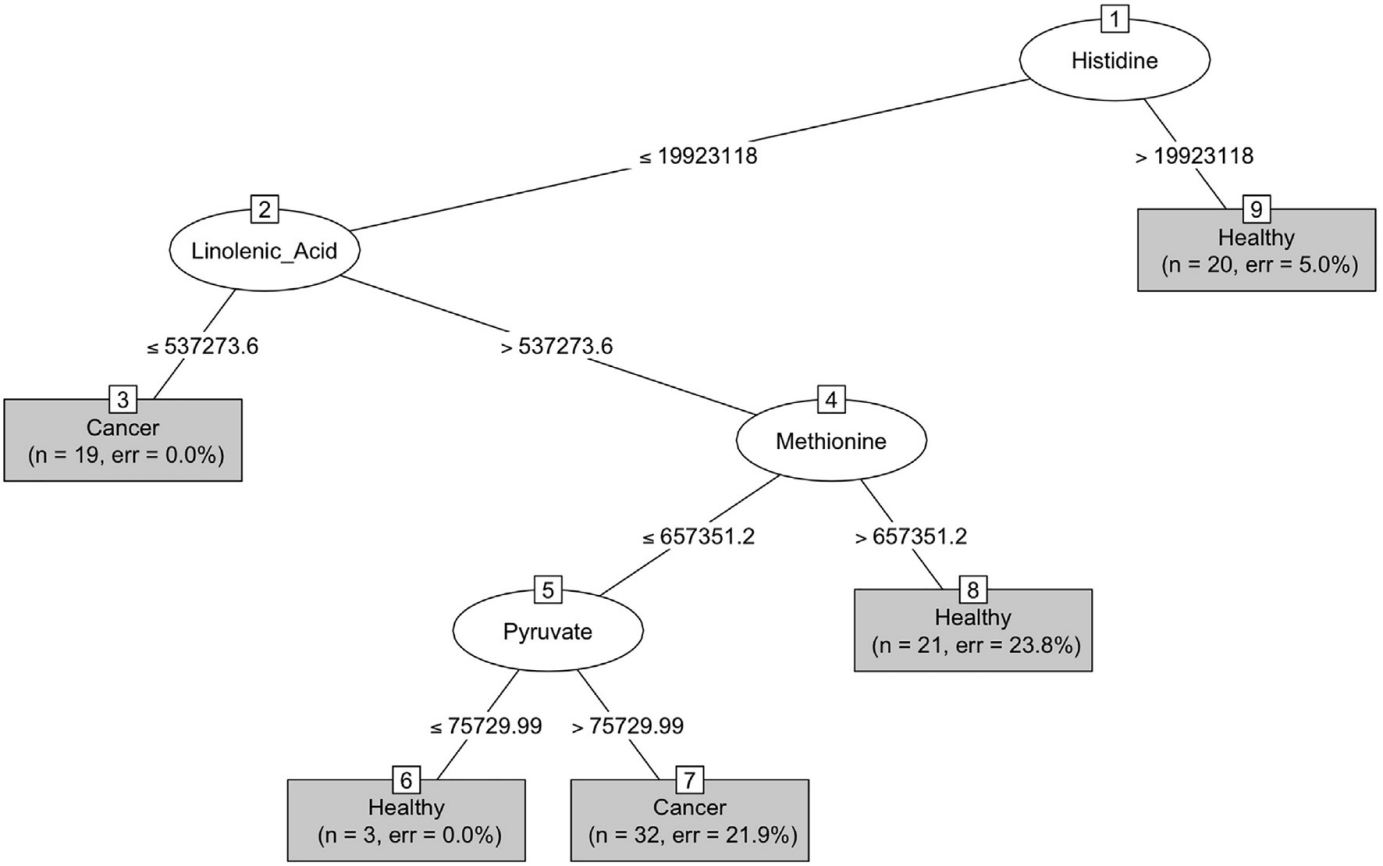

From the initial set of variables, “Acetylcholine”, “Alanine”, “Asparagine”, “Aspartic_Acid” “Biotin”, “Glutamic_acid”, “Glutamine”, “Histidine”, “Linolenic_Acid”, “Lysine” “Methionine”, “MethylSuccinate”, “Pyruvate”, “Tryptophan”, “BMI”, C4.5 detects Histidine, Linolenic_Acid, Methionine and Pyruvate as relevant variables for predicting CRC.

All the experiments in this worked example were performed using RStudio 1.3.1093, R 4.0.3 and caret package, version 6.0-86.

## 9 Using Semantic Web to connect and to exploit data

**Fig. 3:**
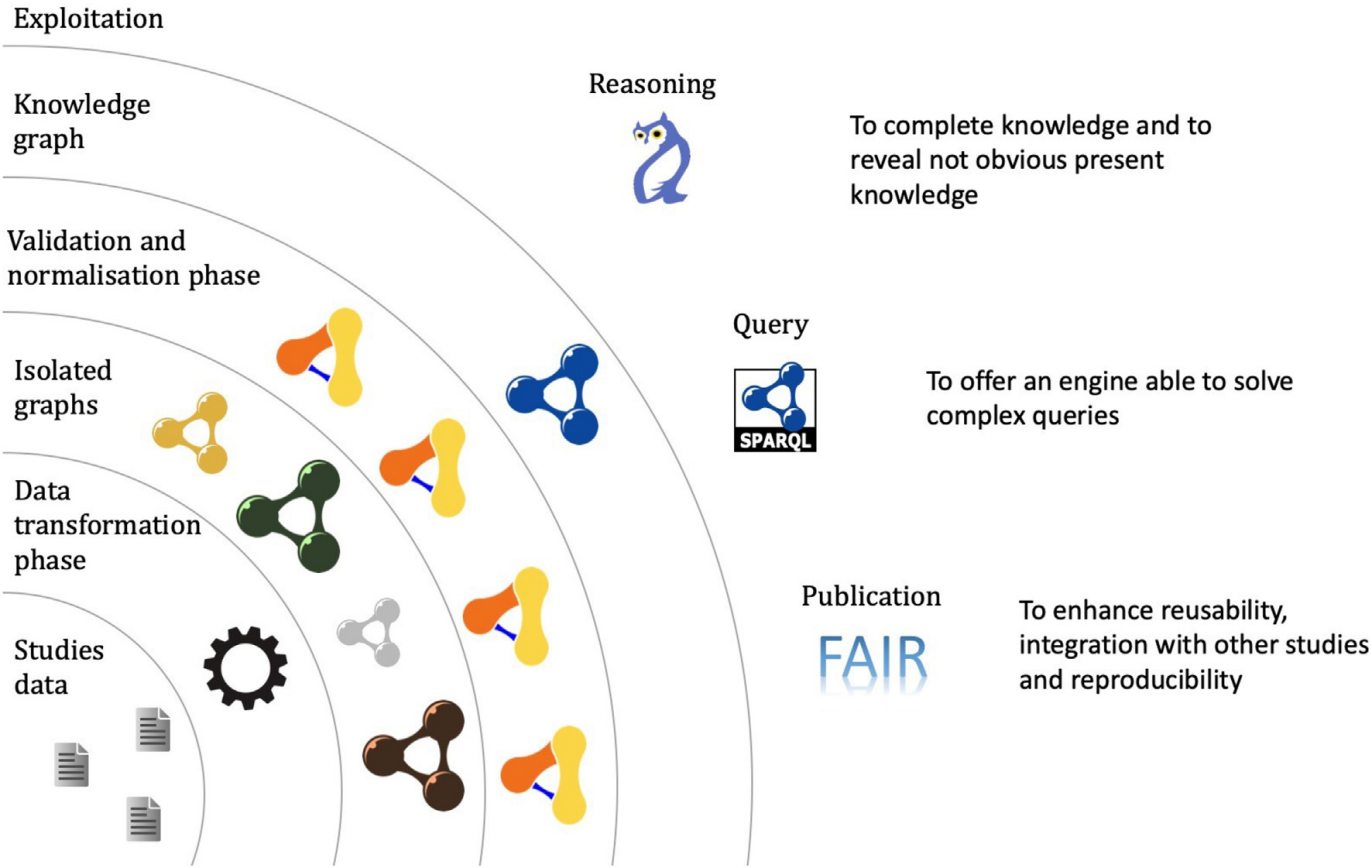
Semantic Web schema and technological stack proposed for microbiota and diet studies. Each concentric circumference represents a layer/process in the technological stack; these layers are independent and can work by themselves. The layer stacking means that an upper layer contains the lower ones and need for them to be complete and coherent. Different coloured graphs represent graphs from different sources, which are not yet integrated. Orange and yellow patterns in the validation phase represent the mechanism of validation and normalization of the aforementioned heterogeneous graphs, which connect to a unique and integrated knowledge graph. (For interpretation of the references to colour in this figure legend, the reader is referred to the web version of this article.)

The Semantic Web vision has supposed a shift of persistence, modelling and interoperability of data [53]. Being able to represent entities unambiguously, link them and integrate different data-sources in a single representation, has enabled a new set of semantic-aware applications. These computer science advances are ready to be applied to different fields. Specifically, in the bio-computational field, some works have explored its use i) to describe human and mouse genes [54] ii) to offer a platform that eases the consumption and curation of genome data [55] iii) to integrate different drug data-sources [56] iv) to provide a platform to analyse the course of diseases [57]. Therefore, we envisage next challenges using Semantic Web technologies to model and to exploit data from nutrition and microbiota interaction studies (Fig. 3).

One of the main problems facing the exploitation of data from these type of studies is the existence of many heterogeneous data-sources with their own data models that cannot be integrated easily with others. This issue prevents obtaining conclusions of the joint-analysis of data from different studies. To alleviate this problem, some ontologies were proposed which ensure that all data providers are talking about the same domain [58]. For example, FoodOn [59] for data integration of food traceability and quality control is a very specific ontology that offers a great basis for reusability. In contrast, ONS [60] is a general ontology for nutrition studies that can be tuned with specific elements if necessary. Alongside the creation of well-defined ontologies, there arises the need for tools able to migrate non-semantic data to these new semantic standards. Recent development of heterogeneous data mapping tools in the Semantic Web has supposed a new paradigm in knowledge graph creation methodologies [61] offering reusability, maintainability and a better user-experience. The use of these tools can deliver a faster migration of non-semantic datasets to a knowledge graph in which all desired studies can be integrated. This will offer the possibility to analyse all data together, make it accessible, and preserve it for future uses, which is in keeping with FAIR (Findable, Accessible, Interoperable and Reusable data) principles [62].

Although a well-defined ontology can enable interoperability and integration of different datasets, we must also ensure that different pieces of data follow the same shape, which will derive in a cleaned and normalised graph and, therefore, an easier one to query. The use of Resource Description Framework (RDF) [63] validation technologies was explored in Fast Healthcare Interoperability Resources (FHIR) specification [64] to not only validate data but to share data models among humans and machines [65]. Therefore, using ontologies, we can define the meta-knowledge of the domain, e.g., the category’s relationships between different mutagens, nutrients or bacteria; using RDF validation techniques we can ensure certain rules, e.g., that a value is between certain limits or that a nutrient has a certain number of attributes.

Once various datasets are converted, validated—using the aforementioned techniques—and their semantics defined using a proper ontology, new results could be delivered. Thanks to ontology axioms it is possible to generate inferences on pre-existing knowledge in order to reveal non-evident and underlying content, which could be obviated [66]. For example, if we define *Bacteroides fragilis* we know that it also belongs to the categories *Bacteroides* (genus), Bacteroidaceae (family), Bacteroidales (order), Bacteroidia (class) and Bacteroidetes (phylum); however, this information is not evident for a machine. Thus, the inference system will fill these upper categories, so all data is complete and can be easily integrated. In addition, the graph data model used by RDF enables a different data modelling—in contrast with the normally used tabular form—, that by means of SPARQL—the advocated RDF query language—could reveal new relationships previously obviated [67]. This simplifies the modelling of the former example in which we have multiple categories, and consequently we wish that *B. fragilis* were shown when asking for a Bacteroidetes, and a Bacteroidaceae, among others. Doing the same modelling in tabular form would imply considerably more complicated structures that can be error-prone.

Finally, this methodology offers the possibility to not only improve analysis techniques and discover hidden content but also to transfer part of this knowledge and make it accessible for the public. The emergence of projects as Wikidata [68] enables the creation of general-purpose knowledge graphs integrating data that could be interesting for the entire world and that is curated by users. It is possible, by taking advantage of proposed conversions, to publish interesting conclusions of involved studies in the so-called semantic eScience [69]. This approach may be employed for the achievement of FAIR principles but also to achieve a transference and dissemination effort, which could lead to a relief in the ongoing reproducibility crisis [70].

## 10 Summary and outlook

The net exposure to dietary toxic compounds, and the intestinal genotoxicity generated, depends on the intake and time of consumption and on their interaction with the IM and global diet. The IM of individuals with CRC differs from that of healthy people, but studies relating the consumption of carcinogens with adverse early shifts of microbiota (either beneficial adaptive or adverse changes) are very scarce. The complexity of data and the several variables potentially affecting these interactions may hinder the interpretation of the studies. In this context, the application of ML to the data obtained in subclinical and precancerous stages of the intestinal mucosa could help to analyse the risk for development of CRC associated to the intake of carcinogens as a function of diet and microbiota profiles. Moreover, the use of the recently developed Semantic Web approaches could improve data accessibility and management, contributing to evidence of new interactions among carcinogens, microbiota, and diet (Fig. 1).

## CRediT authorship contribution statement

**Sergio Ruiz-Saavedra:** Writing - original draft. **Herminio García-González:** Writing - original draft. **Silvia Arboleya:** Writing - original draft. **Nuria Salazar:** Writing - original draft. **José Emilio Labra-Gayo:** Writing - original draft. **Irene Díaz:** Writing - original draft. **Miguel Gueimonde:** Writing - original draft. **Sonia González:** Writing - original draft. **Clara G. de los Reyes-Gavilán:** Writing - original draft.

## Competing financial interest

The authors declare no competing financial interest or personal relationships that could have influenced the content of this article.
